# Editorial for Special Issue “Microorganisms for Environmental and Industrial Applications”

**DOI:** 10.3390/microorganisms6030062

**Published:** 2018-07-02

**Authors:** Anna H. Kaksonen

**Affiliations:** 1 CSIRO Land and Water, 147 Underwood Avenue, Floreat 6014, Australia; anna.kaksonen@csiro.au; 2 School of Biomedical Sciences, University of Western Australia, 35 Stirling Highway, Crawley 6009, Australia

Microorganisms play an essential role in the biogeochemical cycling of elements, and therefore they hold potential for various environmental and industrial applications. Although their importance for bioremediation and maintaining ecosystem health has been well established, there is plenty of room for increasing our understanding on their capabilities and innovating new biotechnical applications. This special issue of Microorganisms provides a platform to discuss new insights into environmental and industrial applications of microorganisms, including but not limited to ecology, diversity, physiology, detection methods, and processes. The thematic areas that are covered by the seven papers of this special issue include microbial perchlorate reduction and reductive dechlorination, biodegradation of organic contaminants in unconventional natural gas extraction sites, microbial tellurite and tellurate reduction, biotechnical mine water treatment using biological sulfate reduction, and biogeochemical phosphorus cycling in agricultural soils. The authors have utilised a variety of analytical chemistry and molecular approaches, such as genomics, transcriptomics, and proteomics to characterise the structure and function of microbial communities.

The review that was authored by Wang and Coates [1] is dedicated to biotechnical applications of microbial perchlorate reduction. Specifically, this paper presents an overview of the emerging biotechnological applications of perchlorate respiration as well as the organisms and enzymes catalysing the reduction. Examples of perchlorate bioremediation systems that are discussed in the paper include *in situ* and *ex situ* bioreactors, which utilise inorganic or organic electron donors, and bioelectrochemical systems with cathodes as electron donors.

As reported by Heavner et al. [2], two of the globally most common groundwater contaminants are perchloroethene (PCE) and trichloroethene (TCE), which can be successfully treated using *in situ* bioremediation [2]. For their paper, Heavner et al. [2] utilised genome-wide transcriptomics and proteomics to identify most highly expressed enzymes relevant for organohalide respiration in a bioaugmentation culture. They then used quantitative reverse-transcriptase polymerase chain reaction (qRT-PCR) as a targeted approach for quantifying putative biomarker transcript copies in the consortium and detected a decline in the biomarkers when the culture was exposed to oxygen stress in continuously-fed, pseudo-steady-state reactors. 

Other concerns of groundwater contamination, as reported by Santos et al. [3], are exogenous hydrocarbons and volatile organic compounds that pollute groundwater as a result of unconventional oil and natural gas extraction activities. In their work, Santos et al. [3] used matrix-assisted laser desorption/ionisation-time-of-flight-mass spectrometry (MALDI-TOF MS) to identify cultivable organic-degrading bacteria in groundwater close to unconventional natural gas extraction. Moreover, they demonstrated the ability of *Pseudomonas stutzeri* and *Acinetobacter haemolyticus* to degrade toluene and chloroform and suggested that these species could be utilised for *in situ* bioremediation.

Maltman et al. [4] explored biological tellurite and tellurate reduction by the aerobic anoxygenic phototroph *Erythromonas ursincola*, and for the first time, purified and characterised a constitutively expressed membrane associated enzyme responsible for the tellurium (Te) oxyanion reduction. This finding may help to further develop environmentally friendly bioremediation methods for these highly toxic substances.

Biological sulfate reduction holds great potential for biotechnical mine water treatment due to the possibility of combined removal of sulfate, metals, and acidity. In this special issue, Bomberg et al. [5] characterised microbial community structure and function in three ethanol-fed laboratory-scale sulfate-reducing bioreactors that were evaluated for mine water treatment. Based on quantitative PCR (qPCR), dissimilatory sulfate reducers comprised only 1–15% of the bacterial communities, although sulfate reduction efficiency of up to 50% was achieved. Based on high throughput amplicon sequencing, the diverse microbial communities also contained archaea and fungi. 

In another paper of this special issue, Rezadehbashi and Baldwin [6] characterised the sulfate-reducing microbial communities in a number of semi-passive pilot-scale bioreactors that are used for metal-removal from mine waters. Through pyrosequencing, they showed that methanogens coexisted with sulfate reducers and competed for available substrates. Depending on the reactor system, various natural organic materials, such as wood chips, manure, pulp mill, and biosolids were effectively used as the organic substrates.

The study by Adhikari et al. [7] focused on the relationship between phosphorus cycling, bacterial biomass, pH, and mineral concentration in agricultural soils. Phosphorus cycling is essential for enabling the availability of phosphorus to plants. The authors proposed a relationship model that is based on the effects of bacterial biomass and mineral concentration on phosphorus circulation activity and used the model to estimate suitable conditions of bacterial biomass, pH, and mineral concentration for phosphorus cycling activity.

In conclusion, hopefully this special issue will serve as a valuable resource for environmental and industrial applications of microorganisms, and encourage others to explore the use of microorganisms for sustainable bioprocessing. Finally, the contributions of each of the authors and reviewers who carefully read and provided constructive comments on the manuscripts are gratefully appreciated.
